# Cancer as a Metabolic Disorder

**DOI:** 10.3390/ijms23031155

**Published:** 2022-01-21

**Authors:** Jones Gyamfi, Jinyoung Kim, Junjeong Choi

**Affiliations:** 1 Yonsei Institute of Pharmaceutical Sciences, College of Pharmacy, Yonsei University, Veritas Hall D 306, 85 Songdogwahak-ro, Incheon 21983, Korea; jones.gyamfi@gmail.com (J.G.); wlal60633@gmail.com (J.K.); 2 Department of Medical Laboratory Sciences, University of Health and Allied Sciences, PMB 31, Ho, Ghana

**Keywords:** cancer, metabolism, glucose, glutamine, fatty acids

## Abstract

Cancer has long been considered a genetic disease characterized by a myriad of mutations that drive cancer progression. Recent accumulating evidence indicates that the dysregulated metabolism in cancer cells is more than a hallmark of cancer but may be the underlying cause of the tumor. Most of the well-characterized oncogenes or tumor suppressor genes function to sustain the altered metabolic state in cancer. Here, we review evidence supporting the altered metabolic state in cancer including key alterations in glucose, glutamine, and fatty acid metabolism. Unlike genetic alterations that do not occur in all cancer types, metabolic alterations are more common among cancer subtypes and across cancers. Recognizing cancer as a metabolic disorder could unravel key diagnostic and treatments markers that can impact approaches used in cancer management.

## 1. Introduction

Despite the progress made in our understanding of cancer, cancer burden has continuously increased. Data from the World Health Organization (WHO) in 2018 indicated that globally, 18.1 million new cases were recorded with 9.6 million cancer deaths [1,2]. In several high-income countries (HICs), cancers have overtaken cardiovascular diseases as the leading cause of death, accounting for twice as many deaths as cardiovascular diseases [1,2]. Our deepening understanding of cancer only seems to reveal the complexity of the disease. The development of cancer involves a multi-step process, evolving from an in situ state and ultimately resulting in a malignant tumor [3]. The invasion and metastasis of cancer cells to nearby or distant organs accounts mostly for all cancer-related morbidity and mortality [4]. Advances in molecular and cell biology techniques have helped unravel key aspects of how neoplastic cells progress through carcinogenesis and acquire their metastatic ability. However, several questions pertaining to carcinogenesis, and metastasis remain unanswered. These insights into the biology of cancer have not translated into effective treatments or even effective long-term management. To date, management of metastatic cancers remain a major challenge as it was 40 years ago [4]. Statistics from the American Cancer Society indicate that 569,490 people died of cancer in the United States in 2010; similarly, in 2002 cancer deaths were estimated to be 555,500 and in 2020 606,520 deaths [5]. These statistics are alarming and indicate the slow pace of progress in cancer management.

The emergence of immunotherapy has opened a new avenue of treatment and offered an opportunity to reduce cancer deaths. However, the long-standing challenges that faced targeted therapy (i.e., resistance and disease relapse) similarly pose a challenge to immunotherapy [6,7]. The explosion in whole genome or exome sequencing of cancer has enhanced our knowledge of genomic changes that occur in cancer but has not translated into the identification of effective targets for therapy. If anything, the advancement of cancer sequencing has compounded the complexity of cancer. Several mutations and genomic alterations are highlighted daily and touted as potential targets for therapy, but targets highlighted from these studies as breakthroughs have not led to transformative therapeutic options. The slow rate of progress in cancer treatment raises great concerns particularly with the significant investment made into research. The major success in several cancers is attributed to improved diagnosis at an early stage, occasioned by enhanced awareness of contributing factors and avoidance of major risk factors [4,8]. These factors are in no way linked to advances in systemic metastasis management [4].

Our progress and approach to cancer management over the years is based strongly on the theory that cancer is a genetic disease, characterized by a sequence of mutations and genomic alterations in cells leading to the acquisition of the cancer phenotype. This theory serves as the basis for the cancer genomic sequencing era. With the promise that the identification of mutations and genetic alterations common to cancers will result in the development of new drugs. However, it is fair to say that this promise has not fully materialized. The recent success of immunotherapy was not steered by the genetic theory of cancer. Thus, it is time that other theories about the origin and basis of cancer development be given much attention. The theory that cancer may be a metabolic disease has lingered for years, and recent evidence provides strong support for this theory. In this review, we seek to provide information on scientific evidence supporting the metabolic theory of cancer, challenges of the theory, and opportunities associated with this theory even in an era of cancer immunotherapy.

## 2. Cancer as a Genetic Disease

Over the years various models have been suggested to explain the complexity of cancer. In the landmark paper by Hanahan and Weinberg, the authors highlighted key features characterizing cancers that have widely been accepted [9,10]. Their model offered strong support for the genetic basis of cancer—the idea that cancer is a disease caused by the accumulation of mutations, epigenetic changes, and genetic alterations in key genes that regulate cell replication, cell division, cell metabolism, and cell growth. These genes are classified as oncogenes and tumor suppressors based on their effect following mutation. The genetic model for cancer development, although not complete, has offered key insights into the genetic events governing cancer initiation, progression, metastasis, response to therapy, and the development of drug resistance [9,10,11]. Support for this theory is widespread, with mutation in certain genes identified in a wide range of tumor types. The era of sequencing has resulted in a catalogue of the various genes mutated in cancers. Presently, over 1000 genes have been linked to cancer and are classified as cancer-associated genes (~250 oncogenes, ~700 tumor suppressors) [12]. The Knudson’s “two-hit” hypothesis postulates that both alleles of a tumor suppressor gene require genetic silencing to cause phenotypic change. Thus, applying the Knudson’s hypothesis to the identified cancer-associated genes translate to over a million potential cancer genotypes [12,13]. How does one disease possibly translate to a million genotypes? How do we successfully manage or treat a million different diseases? This has been a major drawback for the genomic theory of cancer.

The advancement of genomic sequencing has generated enormous genetic data that have further compounded this dilemma. These comprehensive genomic sequence studies in approximately 1 million tumor samples have identified more than 2 million coding point mutations, >6 million noncoding mutations, >10,000 gene fusions, ~61,000 genome rearrangements, ~700,000 abnormal copy number segments, and over 60 million abnormal expression variants [12,14]. In a recent study, whole genome sequencing of tumor samples compared to adjacent normal tissues contained 10,000–50,000 unique single nucleotide variants [12,15]. These studies basically reveal that tumor cells are characterized by millions of genetic alterations. With advancement in sequencing technologies and with the application of single cell sequencing to cancers, our knowledge of the genetic alterations characterizing tumor cells is bound for an explosion. Using these genetic characterizations of tumors to identify targets and develop tumor-specific drugs appears to be a daunting task. Our interest in cancer cannot just be about elucidating the genetic alterations characterizing tumors but should also focus on how these can be translated into effective therapies for cancer management.

The stepwise mutational events governing cancer development are strongly supported in some cancer types than others. Childhood cancers are usually devoid of mutations that characterize adult tumors [16]. While certain mutations are common across several cancers, others appear exclusive to specific cancer types. These discrepancies in the genetic theory of cancer are yet to be resolved. In colon cancer, the sequential mutational events that characterize its development have been extensively studied. This was demonstrated experimentally using sequential CRISPR/Cas guided knockout of the adenomatous polyposis coli (APC) gene, tumor protein p53 (TP53) gene, Kirsten rat sarcoma virus (KRAS) gene, and SMAD family member 4 (SMAD4) gene in intestinal stem cells resulting in mutant tumors with invasive carcinoma when xeno-transplanted into mice [17]. Studies like this provide strong evidence that genetic alterations in genes are key drivers of cancer development. However, unlike colon cancer, such sequential genetic events in genes resulting in cancer cannot be shown for other cancer types. Studies looking to characterize the mutational events in cancers usually show a continual increase in the mutational burden of tumors as they progress into advanced form but do not usually identify genes that are exclusively responsible for each step-in tumor progression. How cells with hundreds of mutations and genomic rearrangements survive and obtain an advantage over healthy normal cells is quite unthinkable. In all our understanding of genetics, this concept appears contrary to the expected, as accumulating mutations should disadvantage cancer cells. Unless the resulting mutations are the outcome of an underlying defect.

Another strong piece of evidence of cancer as a genetic disease comes from the presence of mutations in the genome of cancer cells that do not occur in nearby normal tissues. Despite the hallmarks of cancer being shared by all cancers, not a single gene mutation is linked to all cancers. The gene commonly mutated in most types of cancers is the TP53 gene [18,19]. Despite its high frequency, TP53 mutations are not found in every cancer. In several cancers (i.e., ovarian, esophageal, colorectal, head and neck, larynx, and lung cancer) with high rates of p53 mutations, the rate is about 38–50% [18,19]. The mutational rate of p53 is even lower in other cancers (i.e., primary leukemia, sarcoma, testicular cancer, malignant melanoma, and cervical cancer) occurring at a rate of ~5% [18,19]. This demonstrates that no single gene mutation is a feature in all cancers. The genetic theory postulates that alterations in different sets of genes ultimately result in the cancer phenotype. This explanation is not entirely supported by our current understanding of most genetic disorders where mutations in very different set of genes yield the same phenotypic outcome. Could the mutations that occur in cancers be an outcome of a deeper cause and not the main cause of the tumor. Subsequently, targeting and treating these mutations are just a surface approach. If this theory is true, then there would be a need to find the originating events and target that for therapy. This concept is not farfetched, as a recent study detailing the functions of commonly identified tumor suppressors and oncogenes linked their function to key roles in cellular metabolism [20]. The unlimited mutations in cancer-associated genes affect three main metabolic pathways: the aerobic glycolytic pathway, the glutamine catabolic pathway, and one-carbon metabolism [21]. These genetic alterations create an altered metabolic state allowing cancer cells to generate the large quantities of macromolecules (amino acids, nucleotides, and fatty acids) and metabolic intermediates required to fuel rapid cell growth and division [21]. Thus, could cancer be essentially a metabolic disease?

## 3. Cancer as a Metabolic Disorder

In 1927, Otto Warburg reported that cancer cells manifest a unique metabolic phenotype, characterized by enhanced consumption of glucose compared to normal cells. This phenomenon has become known as the “Warburg effect” [21]. Warburg’s observations led to the notion that cancer was a metabolic disease, an idea that was widely supported until the 1970s when the concept of cancer as a genetic disorder emerged [22]. Recently, the idea of cancer as a metabolic disorder has re-emerged and drawn much attention. Ironically, this growth is supported by enhanced sequencing technologies coupled with increased genetic data and an increased accessibility to metabolomics. The progress in these areas have led to the discovery of oncometabolites; endogenous cellular metabolites that accumulate in tumors sustaining tumor growth and metastasis. The discovery of 2-hydroxyglutarate as an oncometabolite in high concentrations in gliomas led to the discoveries of many other oncometabolites in different cancers [23,24]. The oncometabolite 2-hydroxyglutarate functionally modifies histone methylation patterns, alters differential gene expression, and results in carcinogenesis [25]. Among the recently characterized oncometabolites are fumarate in renal cell carcinoma; sarcosine in prostate cancer; glycine in breast cancer; asparagine in leukemia; choline in prostate, brain, and breast cancer; lactate, glucose, glutamine, and serine in several cancers [26,27]. The increase and accumulation of oncometabolites in cancer is linked to its need to sustain aerobic glycolysis, glutaminolysis, or one-carbon metabolism [26,27]. The discovery of oncometabolites provides evidence for the metabolic state in cancer, linking the emergence of cancer to disturbances in energy production.

Earlier, support for cancer as a metabolic disorder, proposed that cancer arose from defects in energy production through oxidative phosphorylation (OxPhos) in the mitochondria [3]. OxPhos generates most of the energy needed by cells; hence, any defect in the number, structure, and function of mitochondria will alter the energy production in cells [3]. Gradually, this defective energy production results in the replacement of insufficient respiration with fermentation for energy production, resulting in the activation of pathways that results in neoplasia [3,4], hence, aerobic fermentation of lactic acid (Warburg effect) being the most common pathological phenotype of cancers. Recent evidence also indicates that tumor cells can use mitochondrial substrate-level phosphorylation as another fermentation pathway to compensate for defective respiration [3,4]. Mitochondrial substrate-level phosphorylation provides evidence previously missing in Warburg’s theory [3,4]. Defective OxPhos with a compensatory reliance on fermentation for energy produces reactive oxygen species (ROS) that are both mutagenic and carcinogenic [3,4]. Thus, according to the metabolic theory of cancer, the somatic mutations and all other hallmarks of cancer are downstream epiphenomenon occurring from the initial disturbances of cellular energy metabolism [3,4].

A well-known feature of almost all cancers is their high uptake of 2-deoxy-2(18F)-fluoro-D-glucose [28,29]. A feature that is exploited for diagnosis of cancer by positron emission tomography (PET) scans and clearly a metabolic feature. This technique relies on the enhanced dependence of cancer cells on glucose and glutamine for diagnosis, and clearly indicates that both early- and late-stage cancers are characterized by this metabolic feature. This feature is not limited to only a subset of cancer but occurs in almost all cancer types irrespective of the accompanying genetic changes. This feature offers a key insight into cancers: that looking for distinct metabolic events could be useful in cancer diagnosis. Several key metabolites identified in cancers (e.g., acetate, lactate, serine, sarcosine, asparagine, or choline) can be screened in blood, saliva, breath, or urine [12]. Recent studies of metabolites in colonic polyps and early-stage pancreatic cancer demonstrate the potential role of metabolites as biomarkers [30,31]. Ironically, although cancer has been deemed a genetic disease for decades and genetic alterations have continuously been highlighted in cancers, no definitive genetic screening is available for cancer. It is astonishing that not a single gene is known that has mutated across all cancers, yet cancer is considered a genetic disease; however, a metabolic feature observed in >90% of cancers is still overlooked. Metabolite screening holds great potential for future early diagnosis and pre-cancer screening and would be fast and cost-efficient. This clearly reveals the metabolic nature of cancers and provides credence for the metabolic basis of cancer. As indicated earlier, much of the success in cancer management comes from early cancer detection and cancer metabolite screening would be valuable in this aspect.

**Figure 1:**
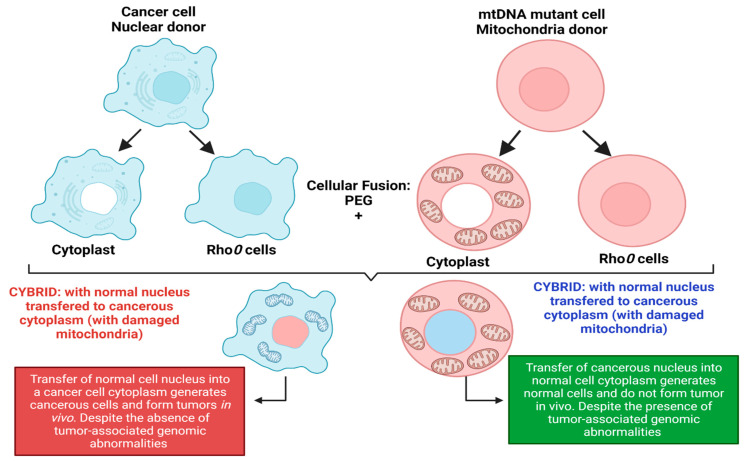
Nuclear–cytoplasmic studies (modified from the nucleus and mitochondria in the origin of tumors as previously described by Seyfried, 2012d; Seyfried et al., 2014). These studies involved the replacement of damaged mitochondria with normal mitochondria or the replacement of the nucleus of a cancerous cell with a normal cell nucleus. If cancer originates from a damaged nucleus, replacement with a healthy nucleus should suppress tumor growth. However, if cancer originates from dysregulated metabolism originating from mitochondria dysfunction, its substitution with a normal mitochondrion should suppress cancer. Exchange of the nucleus of a cancerous cell (shown in blue) with a normal cell nucleus (shown in red) generates a cybrid that generates cancerous cells despite the absence of tumor-associated genomic abnormalities. The exchange of the nucleus of a normal cell (shown in red) with a cancerous cell nucleus (shown in blue) generates normal cells with distinct morphology despite the presence of tumor-associated genomic abnormalities. Figure created using BioRender.

More evidence of cancer as a metabolic disorder comes from nuclear-cytoplasm transfer studies (Figure 1). These experiments involve the replacement of damaged mitochondria with normal mitochondrion or the replacement of the nucleus of a cancerous cell with a normal nucleus with the aim of determining if a damaged mitochondria or damaged nuclear serves as the origin of cancer. If cancer originates from a damaged nucleus, its replacement with a healthy nucleus should suppress tumor growth. However, if cancer originates from dysregulated metabolism originating from mitochondria dysfunction, its substitution with a normal mitochondrion should prevent cancer (Figure 1) [3,4]. These experiments involve the use of cybrids, cells generated when cytoplasm from enucleated normal cells is fused with nucleated tumor cells. The generated cybrids thus have a single nucleus but a blended cytoplasm from different cells. Cybrid studies conducted by Koura et al. involved the fusion of whole cancerous B16 mouse melanoma cells and enucleated non-cancerous rat myoblasts [3,4]. The generated cybrids contain healthy mitochondrion from normal cells but damaged nucleus from cancer cells. Interestingly, the reconstituted cybrids evolved distinct morphology and cellular arrangements [3,4]. In the isolated reconstituted clones, tumorigenicity was repressed (Figure 1). After prolonged cultivation of these reconstituted clones, tumorigenicity remerged in some clones [3,4]. These studies provide concrete evidence to support the potential role of normal mitochondria in repressing the malignant phenotype of cancer cells. Other evidence in support of cybrid experiments were provided by Israel and Schaeffer. They demonstrated that nuclear/cytoplasmic hybrids generated from cytoplasts (nucleus absent) malignant cells with nucleus present normal cells produced tumors in 97% of the animals injected [3,4]. Several nuclear–cytoplasmic transfer studies like these provide evidence for the metabolic basis of cancer. These experiments performed in various model systems from studies in rho (ρ) cells (cells depleted of mitochondrial DNA), Lucke frog renal cell tumors, mouse medulloblastoma, and early mouse embryo show that respiratory competent normal mitochondria could suppress tumorigenicity but normal nucleus is incapable of tumor suppression (Figure 1) [3,4].

Probably, the most compelling evidence from cybrids comes from experiments by Jonasson and Harris conducted in human–mouse hybrids [30,31]. Using hybrid clones generated from fusions of diploid human fibroblasts and lymphocytes with the cells of a malignant mouse melanoma, they demonstrated that human diploid cells were as effective as mouse diploid cells in suppressing the malignancy in vivo, indicating clearly that human nuclear genetic materials are not liable for cancer repression [3,30,31]. To validate their findings, human fibroblast cells were irradiated before fusion with melanoma cells [3,30,31]. The generated hybrids led to substantially higher tumor incidence, indicating that repression of tumor formation likely depended on the function of a radiosensitive extrachromosomal element (Figure 1) [3,30,31]. Their findings are remarkable for several reasons, firstly, it indicated that something in normal cytoplasm was responsible for tumor suppression in malignant cells. Secondly, the suppression of tumors was not dependent on human chromosome or nuclear genetic material. Finally, the cytoplasm factor was sensitive to radiation, capable of being destroyed by irradiation leading to the loss of tumor suppression. The ability of radiation to destroy this cytoplasmic factor is consistent with earlier findings by Warburg that radiation destroys mitochondrial respiration. These studies remarkably indicate that the differentiated state of cells is maintained by normal mitochondrial function thereby repressing carcinogenesis. However, damaged/dysfunctional mitochondria enhance dedifferentiation and promote carcinogenesis. Hence, the origin of carcinogenesis depended on the mitochondria health and not on the genetic material in the cell’s nucleus [3,4]. Which begs the question: why is the genetic theory of cancer the predominant theory? These studies highlight the role of the mitochondria and cellular metabolism in carcinogenesis and the need to explore the role of dysregulated metabolism in carcinogenesis. The metabolic theory of cancer is fast gaining roots in the field of cancer studies and generating much excitement. It is evidently clear that the genetic theory generates more complexity for our understanding of cancer, while the metabolic theory of cancer is incredibly simple and holds great promise for cancer diagnosis and treatment.

## 4. Glucose Metabolism in Cancer

Cancer cells have a heightened macromolecule demand to sustain their enhanced proliferation state; thus, there is an increased demand for macromolecule synthesis or importation. In this heightened energy state, cancer cells rely on enhanced glycolysis to generate ATP with the production of lactate irrespective of oxygen availability [32,33]. The Warburg effect is established as a prominent feature of rapidly growing tumor cells and observed in various cancer types irrespective of their tissue of origin [32,33]. This feature has driven much of the conflict in the origin of cancer, i.e., as to whether cancer is a genetic or metabolic disorder. It raises the question of whether the increased reliance on glycolysis is due to the fact of mitochondria dysfunction or occurs following genetic alterations to sustain the proliferative state of cancer cells with healthy mitochondria.

The reliance on glycolysis to meet ATP needs is a highly inefficient process generating only two molecules of ATP compared to OxPhos, which generates 34 molecules of ATP per complete oxidation of glucose [33]. Rapidly proliferating cells demand lots of ATP molecules, hence why the use of glycolysis in cancer for energy production seems highly inefficient and raises the question of why it is a basic feature of cancer cells. To explain this observation in cancer cells, Warburg hypothesized that the increased dependence on glycolysis stems from a dysfunctional mitochondrion in cancer cells [3,4]. Recent research provides evidence of enhanced glycolysis in cancer cells with functional mitochondria [34,35]. These findings indicate that the enhanced reliance on glycolysis may serve other functions, for example, generating glycolytic intermediates that are essential precursors for various anabolic pathways, ribose-6-phosphate, and biomolecules required by cancer cells [36]. For cancer to be considered a metabolic disease, dysregulated metabolism occurring from damaged or insufficient respiration should be present. Without even completely resolving this puzzle, what is evidently clear is that dysregulated metabolism is a key characteristic of all cancer types. Hence, what remains to be answered is whether genetic alternation results in the altered metabolism in cancer or a dysregulated metabolism gives rise to the genetic alteration seen in cancer [37]. The cancer as a metabolic disease supports the later; thus, cancer occurs from dysregulated metabolism. The theory postulates that the metabolic dysregulation of cancer cells leads to upregulation or suppression of genes regulating metabolism in order to remain viable and proliferate [37].

## 5. Glucose Metabolic Reprogramming in Cancer Cells

**Figure 2:**
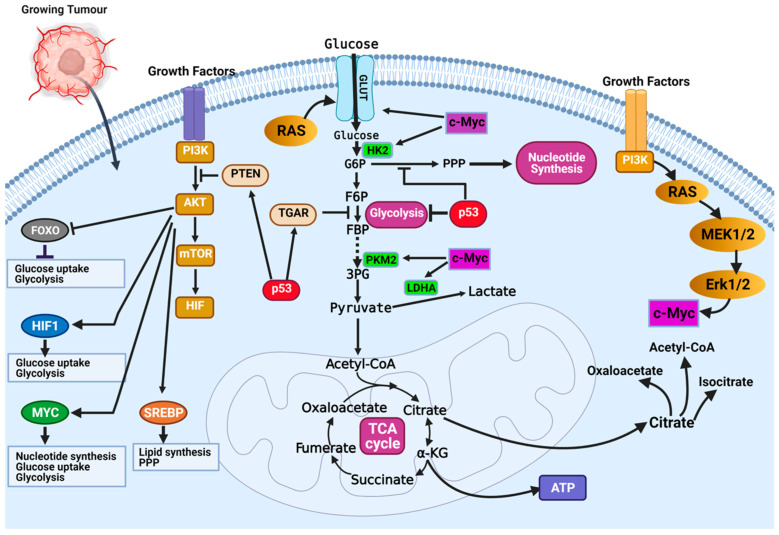
Mechanisms of glucose metabolic reprogramming in cancer. Hypoxia drives metabolic reprogramming in cancer cells. Increased expression of HIF transcription factors activates oncogenes (i.e., Ras, PI3K-Akt, and c-Myc) or inactivates tumor suppressors (p53 and PTEN) to sustain the glycolytic phenotype of cancer cells. Inactivation of the tumor suppressor gene TP53 is a common feature in cancers and contributes to the enhanced dependence on glycolysis. Inactivation of p53 releases repression of glucose transporters (e.g., GLUT1 and GLUT4) and decreases the expression of TIGAR, a glycolytic inhibitor. Activation of growth factor receptors activate the oncogenic PI3K/Akt pathway and activates downstream targets (FOXOs, HIF1a, c-Myc, and SREBP) that contribute to glucose metabolic reprogramming in cancer cells. Figure created using BioRender.

The most dysregulated metabolic pathway in cancer is the glucose metabolism pathway (Figure 2). Glucose metabolic reprogramming occurs across cancer types; however, the specific mechanisms involved in glucose metabolic reprogramming differ among cancer types and even within cancers of the same origin. Central to glucose metabolic reprogramming are changes in the expression of enzymes involved in glucose metabolism (Figure 2) [38]. For a growing tumor, oxygen and nutrient supply is a challenge owing to the tortuous nature of the blood vessels formed in the tumor; the limited supply of oxygen creates a hypoxic environment. Cancer cells subsequently induce the expression of hypoxia-inducible factor 1 and 2 (HIF-1 and HIF-2), transcription factors that regulate the expression of genes during hypoxic conditions [39,40]. Under normoxia, HIF-1α is degraded following oxygen-dependent hydroxylation by prolyl hydroxylase 2 (PHD2) and their recognition by von Hippel–Lindau tumor suppressor (VHL) [39,40]. In hypoxia, VHL-mediated degradation of HIF-1α does not occur, allowing them to accumulate and dimerize with HIF-1β and localize in the nucleus. In the nucleus, HIF-1 dimers regulate the expression of target genes by binding to the hypoxia response element (HRE) sequence of target genes [39,40]. The chronic hypoxic environment in many cancers means HIF-1 becomes constitutively activated to regulate the expression of glycolytic enzymes. Increased HIF-1 expression increases the expression of glucose transporters GLUT1 and GLUT3, enhancing glucose uptake, hexokinase 2 (HK2) to phosphorylate, and commits glucose to the glycolytic pathway (Figure 2) [39,40]. This enhanced glycolytic phenotype of cancers enhances cancer cell migration and invasion, induces angiogenesis, and influences therapeutic response.

Oncoproteins that commonly mutate or genetically alter in cancer cells, including the oncogenic KRAS, oncogenic BRAF, and activated PI3K/AKT, have been shown to result in glucose metabolic reprogramming in cancer cells (Figure 2) [41]. The PI3K/Akt signaling pathway is a convergence point for several tyrosine kinase receptors activated in cancer. The PI3K/Akt pathway is a master regulator of glucose uptake. Activated PI3K/Akt increases the expression of GLUT1 and its translocation to the cell surface [41,42]. Akt also potentiates the activity of the hexokinase which phosphorylate glucose and prevent their efflux from cells. Downstream of the PI3K/Akt pathway is the transcription factor c-Myc. The c-Myc transcription factor (the master transcriptional regulator of metabolism) is overexpressed in ~70% of human tumors. C-Myc dimerizes with c-Myc-associated protein X (Max) to form a heterodimer that transcriptionally regulates the activity of genes that regulate apoptosis, cell growth, and metabolism [33,43]. In contrast to normal cells, where c-Myc expression is induced by growth factor stimulation, c-Myc expression is constitutively activated in cancer cells [33,43]. This increased c-Myc expression promotes energy production and biomolecule synthesis (key requirements in rapidly proliferating cells) by activating target genes involved in glucose transport (GLUT1) and lactate efflux (MCT1) (Figure 2) [33]. Enhanced glycolytic pathways and MYC expression generate glycolytic intermediates that are utilized in other metabolic pathways. Interestingly, HIF proteins can also collaborate with c-Myc to enhance the metabolic advantages of cancer cells. Specifically, HIF-2α stabilizes the c-Myc–Max complex and potentiates their transcriptional regulation of target genes [33]. Alternatively, in normal cells, HIF-1α acts opposite to HIF-2α by binding to Max, and HIF-1α renders c-Myc inactive. In cancers where c-Myc is overexpressed, its activity is not affected by HIF-1α. The increased expression of c-Myc stabilizes the c-Myc–Max heterodimers and reprograms cancer cell metabolism, protein synthesis, and cell cycle progression [33].

In the year 1993, the transcription factor p53 was touted as the molecule of the year by *Science Magazine*. It was regarded as a key molecule in cancer, and scientists hoped this discovery would open an avenue for cancer cure [44,45]. However, this hope has not directly translated into a treatment option. Regarded as a tumor suppressor, p53 is best known for its function in DNA damage response and apoptosis. Recently, important roles of p53 in regulating glycolysis and oxidative phosphorylation have been reported. Functional p53 decreases the glycolytic rate by inhibiting the expression of glucose transporters (i.e., GLUT1 and GLUT4) and decreasing the levels of phosphoglycerate mutase, the enzyme responsible for converting 3-phosphoglycerate to 2-phophoglycerate in glycolysis (Figure 2) [33]. Wild-type p53 regulates the expression of the tumor suppressor, phosphate and tensin homolog (PTEN), that inhibits the PI3K pathway [33]. Inhibition of PI3K leads to decreased activation of Akt1 and HIF proteins, which are essential drivers of glycolysis (Figure 2) [33]. The high frequency of p53 mutation in cancers means it controls metabolic regulation and glycolysis is lost. Cancer cells with mutant p53 have increased expression of glucose transporters, glycolytic enzymes, and activation of AKT and HIF.

Taken together, these findings reveal that multiple growth signaling nodes and key oncogenes identified through genetic studies of cancers remotely facilitate cancer cells’ cellular responses to regulating glucose metabolism. The glucose metabolic reprogramming occurring in cancer cells includes increased expression and translocation to the plasma membrane of GLUT1 and other glucose transporters and increased expression of enzymes involved in glycolysis [41]. This metabolic reprogramming is orchestrated by multiple mechanisms involving oncoproteins and oncogenic transcription factors.

## 6. Glutamine Metabolic Reprogramming in Cancer

Along with glucose metabolic reprogramming, cancer cells exhibit a substantial metabolic flexibility including an altered amino acid metabolism. A common metabolic feature in cancer cells is the increased levels of glutamine metabolism commonly referred to as “glutamine addiction” [32,46]. Glutamine addiction refers to the enhanced dependence on glutamine as a catabolic and anaplerotic substrate. The increased demand for glutamine is utilized in cancer cell metabolism. Its precursor, glutamic acid or its salt glutamate, regulate signaling pathways, proliferation, and metastasis in cancer cells [47]. Glutamine is an important nutrient in oxidative metabolism, ATP generation, biosynthesis of biomolecules, redox homeostasis, and the regulation of signal transduction. Glutamine is an anaplerotic substrate for the TCA cycle, generating metabolic intermediates [47,48]. Glutamine is utilized for energy generation and generates carbon and nitrogen for biomass accumulation in rapidly proliferating cells such as cancer cells, lymphocytes, and enterocytes of the small intestine [33,46].

In cancer cells, the increased demand for glutamine is achieved through membrane transporters. Glutamine is imported into cells by membrane glutamine transporters, including SLC1A5 (also known as ASCT2), SLC7A5, SLC38A1, and SLC38A217, with SLC1A5 being the most studied [46,49,50]. Imported glutamine can be utilized for biosynthesis of nucleotides, NADPH, and antioxidant. The first step of glutamine utilization is its conversion to ammonium ion and glutamate by cytoplasmic/mitochondrial glutaminases (GLS) [51]. The mitochondrial enzyme glutamate dehydrogenase (GLDH; encoded by the highly conserved and more broadly expressed GLUD1 or the hominoid-specific GLUD2, collectively termed GLUD) convert glutamate to α-ketoglutarate. Alternatively, glutamate conversion to α-ketoglutarate can be achieved through several non-ammonia-producing aminotransferases [46]. The α-ketoglutarate produced from this process enters the TCA cycle to generate ATP through the production of reduced molecules NADH and FADH_2_ [48]. In glucose scarce periods, cells utilize glutamate dehydrogenase (GDH) to produce α-KG without requiring an amino acceptor. This means in periods of glucose abundance, glutamate dehydrogenase is dispensable; however, in periods of glucose deprivation glutamate dehydrogenase is required for cell survival [48,52]. The reliance of cancer cells on glucose and glutamine allows them to meet their metabolic needs and maintain a steady supply of carbon-skeleton molecules or metabolic intermediates that can be utilized for anaplerosis to generate macromolecules to meet their cellular demands.

## 7. Mechanisms of Glutamine Metabolic Reprogramming

**Figure 3:**
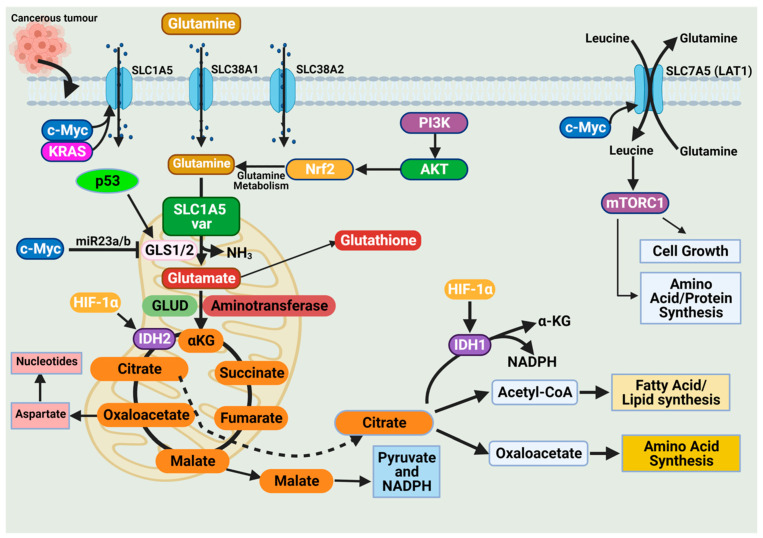
Glutamine import occurs through glutamine transporters and is utilized in various anabolic pathways such as nucleotides, lipid synthesis, and amino acid synthesis. Cancer cells exhibit a dependence on glutamine (i.e., glutamine addiction), and glutamine catabolism in the mitochondria generates metabolic intermediates to sustain cancer cell biomass. The dependence of cancer cells on glutamine is sustained by alterations in oncogenes and tumor suppressors. Expression of the glutamine transporter SLC1A5 is increased in various cancers to enhance their dependence on glutamine. Activation of the oncogenes KRAS and c-Myc further enhances SLC1A5 expression. The oncogene c-Myc indirectly regulates glutaminolysis through inhibition of miR23a/b, a microRNA involved in regulating GLS1/2 expression. The tumor suppressor p53 upregulates GLS2 expression and enhances glutaminolysis. Glutamine activates the mammalian target of rapamycin complex 1 (mTORC1), which functions to support cancer cell growth. Figure created using BioRender.

The high demand for amino acids by tumor cells requires a steady upregulation of selective amino acid transporters. Different tumor types rely on different amino acid transporters to support their growth. Compared to normal tissues, the expression of amino acid transporters is elevated in various tumors. SLC7A5 is an obligatory exchanger that couples the influx of one amino acid substrate into cells with the mandatory efflux of another amino acid substrate (Figure 3) [38,53]. Increased SLC7A5 expression has been reported in many cancer types including triple-negative breast cancer, colon cancer, lung cancer, glioblastoma, and prostate cancer. The main function is to maintain amino acid supply to cancer cells [38,53]. Clearly, the regulation of SLC1A5 expression is an essential activity in cancer cells. Much of this regulation is reported to occur at the transcriptional level. The elevation of HIF transcription factors in the tumor hypoxic environment does not only alter the cellular glucose metabolism. Hypoxia-induced expression of HIF-2α upregulates SLC7A5 expression, allowing for increased importation of glutamine to support the tumor metabolic needs [52,54,55]. In addition, under hypoxic conditions, GLS1 expression is transcriptionally upregulated in an HIF-1α-dependent manner. Hypoxic cancer cells reprogram glutamine metabolism from glutamine oxidative metabolism towards reductive carboxylation utilizing glutamine to generate citrate to sustain proliferation [55,56].

The promoter region of SLC7A5 also contains the canonical binding sites for c-Myc, hence the increased expression of c-Myc in cancer that allows for constitutive expression of SLC7A5. C-Myc and n-Myc control of SLC7A5 expression has been demonstrated in glioblastoma and neuroblastoma, respectively. C-Myc and n-Myc activation induces SLC1A5 expression enhancing glutamine uptake and metabolism. Consensus sequences for c-Myc are found upstream of promoters for different amino acid transporter genes (i.e., ASCT2 (SLC1A5)), allowing c-Myc to directly bind and increase SLC1A5 expression. The increased SLC1A5 expression results in elevated uptake of glutamine and increased catabolic usage. C-Myc is also reported to regulate glutaminase activity. Mitochondrial glutaminase (GLS1) expression is regulated by c-Myc and has gained significant interest in cancer. C-Myc was shown to increase expression of GLS1, enhancing cancer cell proliferation and survival (Figure 3) [57].

Glutaminases involved in glutamine metabolism have altered expression in cancer. While GLS1 expression is usually upregulated in cancers, GLS2 expression is generally repressed [56]. Like SLC7A5, the oncogene c-Myc regulates the expression of key enzymes involved in the glutamine catabolic pathway i.e., GLS1, glutamine synthetase (GLUL), GLUD, and aminotransferases (Figure 3) [52,56,58]. Unlike the direct regulation of SLC1A5 by c-Myc, c-Myc regulation of GLS1 occurs indirectly through transcriptional repression of miR-23a and miR-23b [52,56,58]. MiR-23a and miR-23b are microRNAs known to target the 3’ untranslated regions (UTRs) of GLS1, decreasing expression (Figure 3). Several oncogenic pathways have been implicated in the regulation of GLS activity in a c-Myc dependent manner. The oncogenic transcriptional factor c-JUN is also reported to regulate GLS1 expression [52,56,58]. GLS1 is regulated indirectly through the GSK3α/β pathway modulating the protein stability of c-Myc and c-Jun. The mTORC1/S6K1 pathway positively regulates GLS1 through the eIF4B-dependent control of c-Myc translation [46,56,59]. Post-transcriptional modification of glutaminase is another common regulatory mechanism. Mutations in oncoprotein genes also affect glutamine dependence in cancers. KRAS mutations are frequent events in cancers and are shown to influence cancer cell metabolism. KRAS diverge glucose from the TCA, enhancing glycolysis and glutamine addiction [54,58]. In KRAS-mutated cells, the NRF2 (nuclear factor erythroid 2-related factor 2) pathway reprograms metabolism toward glutamine dependence (Figure 3) [54,58].

These examples indicate the myriad of ways cancer cell metabolism can be altered to depend on glutamine. These examples are only a fraction of the available data highlighting the enhanced dependence on glutamine in cancer cells. This compounding evidence provides support for the metabolic nature of cancer and that commonly identified mutations alter pathways to sustain the metabolic demand of cancer cells.

## 8. Fatty Acid Metabolic Reprogramming in Cancer

A less studied aspect of cancer metabolic reprogramming is lipid metabolism. Recently, attention is being drawn to the roles altered lipid metabolism play in cancer growth [60]. Lipids, also referred to as fats, are building blocks for various classes of lipids comprising triglycerides (TGs), phospholipids, sphingolipids, cholesterol, and cholesterol esters [60]. Regulation of lipid metabolism is key in cellular function and health, and dysregulated lipid metabolism contributes to metabolic disorders, i.e., cardiovascular diseases, obesity, diabetes and, recently, cancer [61,62]. In cancer cells, the requirements for metabolic intermediates for macromolecule synthesis are high, and lipid metabolism serves as an essential pathway for the supply of metabolites [61,62]. Cancer cells strike a balance between lipid anabolism and catabolism, utilizing corresponding signaling networks to generate molecules for membrane formation, energy storage, signaling molecule production, and ATP generation via fatty acid oxidation (FAO) [63]. Extensive research into the role of FA in cancer cell metabolism and tumorigenesis has revealed the dependence of cancer cells on de novo biosynthesis and exogenous FA uptake to support their enhanced proliferative state, particularly in periods of metabolic stress. Recent studies are shedding light on their role in cancer cell metastasis and response to therapy.

The initial step in fatty acid metabolism involves the import of fatty acids into cells. Several membrane-associated fatty acid transporters have been identified and characterized: these included CD36/FAT, FABPpm, and FATPs [62,64]. FATPs are integral membrane proteins with six identified isoforms (FATP1–6). The membrane protein CD36/FAT (88 kDa) is another well-characterized fatty acid transporter that play key roles in fatty acid uptake and β-oxidation. The functions of CD36/FAT have also been linked to angiogenesis, inflammation, and lipid metabolism [65,66]. Functionally, CD36 differs from FATPs in that CD36 is shuttled intermittently between intracellular endosomes and the plasma membrane of cells, allowing it to function in fatty acid uptake and β-oxidation regulation [65,66].

Imported fatty acids in the cytosol are activated to fatty acyl-CoA and shuttled to the mitochondria for oxidation. In the mitochondria, β-oxidation catabolizes fatty acids that produce acetyl-CoA, which fuels ketogenesis and the TCA cycle. The generated reduced FADH and NADH are fed into the electron transport chain [67,68].

## 9. Mechanisms of Fatty Acid Metabolic Reprogramming

A less studied aspect of metabolic reprogramming in cancer cells is their lipid metabolic abnormalities. However, recently, the potential role of altered lipid metabolism in cancer is increasingly being recognized. Deciphering the genetic alterations that characterize fatty acid metabolism has been a daunting task. The number of ways by which fatty acid metabolism can be altered are vast, ranging from upregulation of enzymes that are involved in lipogenesis and upregulation of transporters involved in fatty acid import and enzymes involved in FAO [68,69].

**Figure 4:**
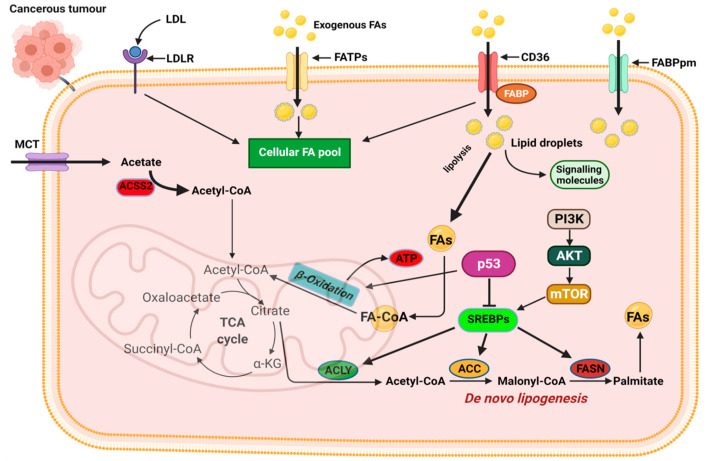
Mechanisms of fatty acid metabolic reprogramming. De novo lipogenesis and exogenous uptake of fatty acid both occur to sustain the altered lipid metabolism in cancer cells. The transporters CD36, FATPs, and FABPpm regulate exogenous fatty acid import. Increased CD36 and FATP expression is reported in various cancers. The oncogenic PI3K/Akt pathway is activated to regulate fatty acid metabolism. Activation of SREBPs is key in de novo lipogenesis and catabolism of imported fatty acids. MCT, monocarboxylate transporter; CD36, cluster of differentiation 36; FAs, fatty acids; FATPs, fatty acid transport proteins; FABPpm, fatty acid-binding protein; ACLY, ATP–citrate lyase; ACSS2, acyl-CoA synthetase short-chain family member 2; ACC, acetyl-CoA carboxylase; FASN, fatty acid synthase; SREBPs, sterol regulatory element-binding proteins. Figure created using BioRender.

The first level of alteration pertaining to fatty acid metabolism in cancer relates to de novo lipogenesis. In various cancers, key regulators of lipogenesis are significantly upregulated including transcription factors. The sterol regulatory element-binding proteins (SREBPs), acetyl-CoA carboxylase (ACC), fatty acid synthase (FASN), and stearoyl-CoA desaturase 1 (SCD1), are among the most reported alterations linked to fatty acid lipogenesis (Figure 4) [68,69]. The increased de novo lipogenesis in cancer cells were demonstrated several years ago when Medes and colleagues demonstrated that cancer tissues relied on de novo lipogenesis to generate fatty acids and phospholipids at levels similar to liver tissues [70,71]. They also noted that exogenous import of lipids occurred in tumor tissues; however, de novo lipogenesis supplied the bulk of lipids required for tumor growth [70,71].

SREBP expression is regulated by intracellular sterol concentrations. In periods of low sterol concentrations, SREBP/SCAP complex translocate to the Golgi complex, and it is cleaved by the membrane-bound proteases (i.e., MBTPS1 and MBTPS2) releasing the transcriptionally active fragment [72,73]. The active fragment containing the DNA-binding and transcriptional activation domains translocate into the nucleus and binds to the sterol-regulatory elements within the promoter of target genes to regulate their activity [72,73]. SREBPs are downstream targets of growth factor signaling pathways that sense and respond to nutrient and cellular energy status [74]. In several cancers, including breast, ovarian, and prostate, dysregulated SREBP activation and expression of target genes have been reported. Activation of the PI3K/Akt pathway in response to growth factor signaling activates SREBPs and its target genes responsible for cholesterol and FA biosynthesis (Figure 4) [74]. SREBP activity is also regulated by AMP-regulated protein kinase (AMPK) regarded as the central sensor of cellular energy. Phosphorylation of SREBP by AMPK inhibits its proteolytic cleavage and activation [74].

Changes in SREBP expression during cancer progression occur with changes in the expression of SREBP-regulated target genes [75,76,77,78]. SREBP1 promotes cancer cell proliferation, migration, and invasion through transcriptional regulation of androgen receptor (AR) gene expression [75,76,77,78]. SREBP1 is linked to fatty acid and phospholipid synthesis, whiles SREBP2 is linked to cholesterol synthesis through its regulation of 3-hydroxy-3-methylglutaryl-CoA (HMG-CoA) reductase (the rate-limiting enzyme in cholesterol synthesis), mevalonate kinase (MVK), and other key enzymes [74]. Several key pathways implicated in tumorigenesis, such as the p53 and PI3K/Akt pathways, activate SREBP2 to promote tumorigenesis. SREBP2-mediated tumorigenesis is reported to occur, in part, by SREBP2-mediated mevalonate metabolism activating the EMT program in cancer cells [77,79]. Significant upregulation of SREBP2 has been reported in prostate cancer, breast cancer, and hepatocellular carcinoma and has been touted as a potential target for therapy [77,78,80]. Oncogenic PI3K or K-Ras activation of mTORC1 is mediated, in part, through SREBPs, inducing de novo lipogenesis to promote tumor growth and proliferation with increased SREBP-2 expression correlating with poor prognosis in cancer patients (Figure 4) [62,78].

The multifunctional polypeptide, fatty-acid synthase (FASN), catalyzes the last step in de novo biogenesis of fatty acids [81]. FASN produces saturated fatty acids sequentially by adding seven malonyl-CoA molecules to one acetyl-CoA to form the 16-carbon palmitate [82,83]. FASN has been studied in various cancers, these studies report FASN overexpression correlates with tumor progression [81,84,85,86,87].

A second level of altered lipid metabolism occurring in cancers is related to lipid uptake from exogenous environments. As our understanding of how the tumor microenvironment influences cancer progression deepens, it is becoming clear that cancer cells acquire and accumulate lipids from their microenvironment and utilize them in various processes to drive their progression. Aside from its role in transporting fatty acids into cells, CD36 has been implicated in a myriad of roles that enhance cancer cell growth, metastasis, and EMT (Figure 4) [88,89]. Importantly, CD36 is linked to metabolic crosstalk between cancer cells and their microenvironment and drives the tumor cell’s dependence on exogenous lipids [89,90]. High CD36 expression has been reported in various cancer types and is correlated with poor prognosis in cancers [88,90]. Mechanistically, CD36-dependent lipid uptake is linked to allowing metastatic-initiating cells to acquire lipids from the extracellular environment and utilize them through FAO to generate ATPs and meet their high energy need [90,91].

A second class of exogenous fatty acid importers are the FATPs. FATP transporters are unusual in that they also express intrinsic very long acyl-CoA synthase (ACSs) enzyme activity that allows fatty acids to be activated for α/β-oxidation in the mitochondrial or peroxisomes (Figure 4) [92]. FATPs are basically involved in the uptake of free fatty acids and esterification of imported fatty acids with CoA to generate acyl-CoA [92]. Studies into the role of FATPs in cancer are limited with few studies highlighting the role of specific FATPs in cancer [93]. Overall, FATPs seem to play an important role in lipid uptake and metabolism. The role of FATPs in cancer, particularly in relation to the import of exogenous fatty acid to enhance tumorigenesis, are just beginning to emerge and further research is required.

Intracellular trafficking of fatty acids is another aspect of altered lipid metabolism commonly highlighted in cancer cells. Fatty acid binding proteins (FABPs) are a class of proteins involved in intracellular shuttling of fatty acids chains, bile acids, and retinoids [94]. The role of FABPs in facilitating intracellular fatty acid transport is known; however, their physiological functions are not fully elucidated [94,95]. FABP5 is the most characterized FABP isoform in cancers. Increased FABP5 expression has been reported in hepatocellular carcinoma, cholangiocarcinoma, and liver, pancreatic, cervical, and breast cancers [96,97,98,99,100]. FABPs are rapidly emerging as essential proteins in fatty acid transport and metabolism in cancer cells. More research is required to understand and fully elucidate the diverse functions of FABPs in tumor cells.

A final step in altered lipid metabolism in cancer relates to alterations that influence lipid catabolism to meet their enhanced energy needs and sustain growth and proliferation. Reprogramming of FA oxidation is a critical feature of cancer cells, and it is the culminating step following the increased de novo lipogenesis and enhanced lipid import from exogenous sources. Lipid metabolism through β-oxidation generates ATP and metabolic intermediates to supplement the metabolic demands of cancer cells. Aside from fatty acid oxidation generating twice as many ATPs compared to carbohydrates, FAO also generates cytosolic NADPH to support biosynthesis (Figure 4). At present, our understanding of the role of β-oxidation in cancer is limited with only a handful of studies reporting increased expression of FAO enzymes in tumors, highlighting the relevance of the pathway to cancer. Current advances in FAO studies are rapidly changing our understanding of the relevance of FAO to cancer. Accumulating evidence indicates the altered FAO activity contributes in several ways to sustaining cancer cell proliferation, survival, stemness, drug resistance, and metastasis [101]. Regulators of the FAO and enzymes catalyzing the various reactions have emerged as potential targets for cancer therapy.

## 10. Conclusions

For a long time, the theory that cancer is a genetic disorder has driven cancer research. Despite all the progress and understanding gained through cancer sequencing, this knowledge has not always translated into relevant treatment options. A prominent characteristic of cancer cells is an altered metabolic state, with alteration in metabolism affecting the glucose, glutamine, and fatty acid metabolic pathways. These alterations generate ATP and metabolic intermediates that sustain the proliferative state of cancerous cells. Metabolic alterations are achieved through several mechanisms, and characterizing these alterations holds promise for identifying targets for diagnosis and therapy. Our understanding of the metabolic reprogramming of cancers continues to grow, particularly with the emergence of oncometabolites and their roles in carcinogenesis, diagnosis, and treatment. Considering cancer as a metabolic disease will open new avenues for research, and more insight will be gained into the metabolic basis of cancer and how we can effectively target these alterations for efficient cancer management.
